# Simulation of developing human neuronal cell networks

**DOI:** 10.1186/s12938-016-0226-6

**Published:** 2016-08-30

**Authors:** Kerstin Lenk, Barbara Priwitzer, Laura Ylä-Outinen, Lukas H. B. Tietz, Susanna Narkilahti, Jari A. K. Hyttinen

**Affiliations:** 1 Department of Electronics and Communications Engineering, Tampere University of Technology, BioMediTech, PL100, Tampere, Finland; 2 Faculty of Engineering and Computer Science, Brandenburg University of Technology Cottbus-Senftenberg, Platz der Deutschen Einheit 1, 03046 Cottbus, Germany; 3 NeuroGroup, Institute of Biomedical Technology, University of Tampere, BioMediTech, PL100, Tampere, Finland

**Keywords:** Simulation, Microelectrode array, Neuronal networks, Human embryonic stem cells, Development

## Abstract

**Background:**

Microelectrode array (MEA) is a widely used technique to study for example the functional properties of neuronal networks derived from human embryonic stem cells (hESC-NN). With hESC-NN, we can investigate the earliest developmental stages of neuronal network formation in the human brain.

**Methods:**

In this paper, we propose an in silico model of maturating hESC-NNs based on a phenomenological model called INEX. We focus on simulations of the development of bursts in hESC-NNs, which are the main feature of neuronal activation patterns. The model was developed with data from developing hESC-NN recordings on MEAs which showed increase in the neuronal activity during the investigated six measurement time points in the experimental and simulated data.

**Results:**

Our simulations suggest that the maturation process of hESC-NN, resulting in the formation of bursts, can be explained by the development of synapses. Moreover, spike and burst rate both decreased at the last measurement time point suggesting a pruning of synapses as the weak ones are removed.

**Conclusions:**

To conclude, our model reflects the assumption that the interaction between excitatory and inhibitory neurons during the maturation of a neuronal network and the spontaneous emergence of bursts are due to increased connectivity caused by the forming of new synapses.

## Background

Human pluripotent stem cells (hPSC), which include human embryonic stem cells (hESC) and human induced pluripotent stem cells and their neural derivatives, have great potential in the fields of neurotoxicity, drug screening, developmental biology, and tissue engineering [1, 2]. Thus, hPSC-derived in vitro neuronal networks can be used as a valuable tool for a variety of purposes, although they have not been studied in such great detail as rodent-derived neuronal cultures [3, 4]. One special aspect of neuronal cultures derived from hPSCs is that their maturation process, when both the cells and the network are maturing, resembles the most primitive stages of human brain formation. Hence, more intensive research is needed to better understand the electrical functionality and maturation of hPSC-derived neuronal cells. Microelectrode array (MEA) experiments are a powerful tool in the study of in vitro neuronal networks [5–8]. MEAs enable the development of neuronal networks to be studied both temporally and spatially. This is also the case with human embryonic pluripotent stem cells-derived neuronal networks (hESC-NN) [6, 9]. Thus, measurements and analyses of the developing human neuronal system at the network level are possible even over long periods, as shown by Heikkilä et al. [6] who used MEAs to follow the neuronal activity of hESC-NN for up to 4 months.

As hESC-NNs mimic the earliest possible human neuronal networks, they most likely differ from in vitro neuronal networks derived from rodent primary cultures. For example, the formation of hESC-NN is a slower process and probably the neuronal precursor cells provide larger capacity for network modulation [6]. Therefore, the development of novel tools for burst and spiking analysis for these hESC-NNs is needed to unveil the temporal and spatial properties of neuronal activity [10]. One major feature of maturing neuronal networks on MEAs is the development of spontaneous bursting activity [5, 11–13] that, according to Heikkilä et al. [6], takes a few weeks from the initiation of the cultures. During this period, the neurons seek connections, make processes, synapses, and modulate their strength [14]. To analyze these changes, one possibility is apply computational models. Computational modeling enables us to analyze the role of various neuronal processes such as axonal length, number, and the strength of the connections between neurons. In silico modeling offers a way to inspect neuronal systems with an artificial system, where all the elements are known and controlled. To the best of our knowledge, there have been no publications on the simulation of the maturation process of hESC-NNs.

The general formation/maturation process of neuronal networks in general has been modeled only in a few papers. Gritsun et al. [15] present a spiking neuronal network model of dissociated rat cortical cells with wiring topology. The model includes approaches for neurite outgrowth, neurite guidance, and mimicking axono-somatic targeting. The authors do not consider any pruning of synapses over time. Kaiser et al. [16] suggest a model of spatial network growth. The model features clusters and the average shortest path, a central topological network measure. However, the model does not consider the role of spontaneous activity in neuronal networks. Furthermore, only a few papers model the morphology during neuronal growth [17–19].

The aim of the present work is to simulate the maturation of hESC-NNs, and thus to evaluate their functioning and network development in different developmental stages in silico. In particular, aspects of the neuronal network development such as the emergence of spontaneous spikes and the development of a burst structure are simulated. In particular, we have concentrated on the development of connections between the neurons and do not consider the spatial distribution or the expansion of the network. Neuronal communication is mediated mainly by synaptic communication; however, there exist other communication paths, like gap junctions [20, 21]. We concentrate on the synaptic pathway similarly as in most neuronal network models [22, 23].

Previously, we built a phenomenological model called INEX (INhibitory-EXcitatory) that was used to simulate neuronal activity recorded from the frontal cortex cultures of embryonic mice using in vitro MEAs [24]. The INEX model is based on inhomogeneous Poisson processes [25] used to simulate neurons that are spontaneously active without external input or stimulus, as observed in MEA experiments. Each neuron has either an inhibitory (negative synaptic strength) or an excitatory (positive synaptic strength) effect on its neighbors. These models of synaptic communications can be considered to include all types of interactions between the neurons. The activity of a neuron depends on its previous spiking history.

Here, the INEX model is used to simulate the developing hESC-NNs on MEAs. The model and its parameters are tuned to mimic the activity measured from in vitro hESC MEA data from six measurement time points during neuronal network activity development and maturation. The activity level is defined as various spike and burst parameters. Thus, the modeled neuronal networks will produce statistically similar spike and burst activity as the in vitro actual neuronal system. Therefore, the main question we aim to answer with the simulations is: Which aspects of the maturation process contribute to the development of stable burst patterns?

## Methods

### Cell cultures

**Fig. 1:**
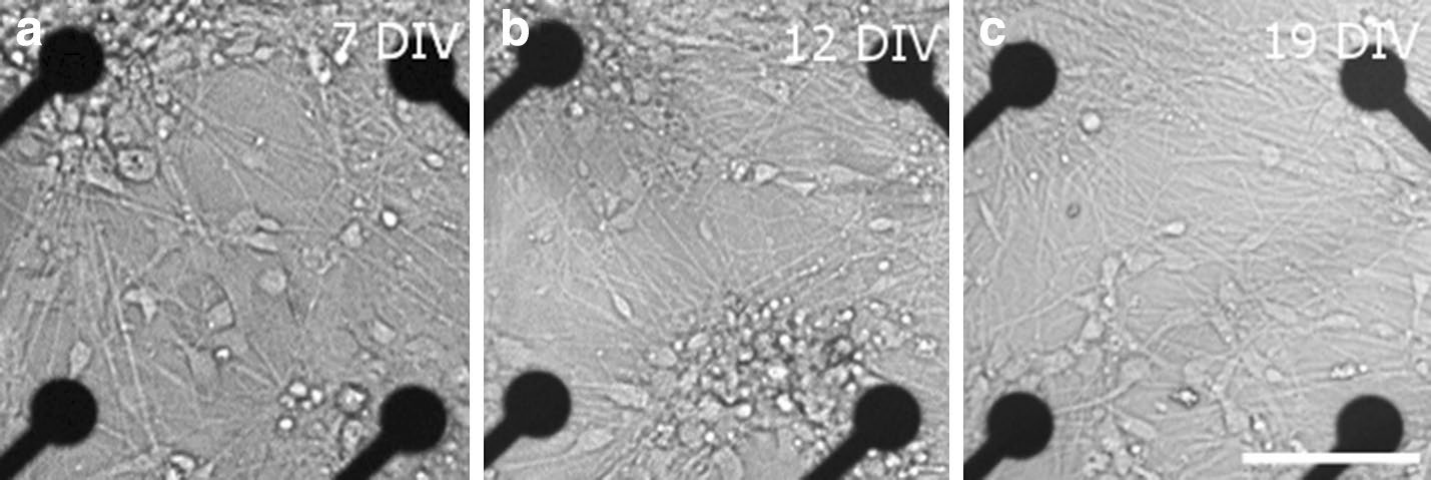
Neuron distribution of dataset #3 (see Table 1) on the MEA for three points in time (**a** 7 days in vitro (DIV), **b** 12 DIV, and **c** 19 DIV). It is clearly visible that the number of neuronal connections increases and the neurons move over time. The *black dots* indicate the MEA electrodes. The *scale* is 100 μm

Human embryonic stem cells (hESCs) [cell lines Regea 08/023 and 11/013] were differentiated into neuronal cells using the previously published method [9] and plated on MEAs as described in Heikkilä et al. [6]. Briefly, cells were differentiated for 8 weeks in differentiation medium containing D-MEM/F-12 and Neurobasal (1:1, both from Gibco Invitrogen, Carlsbad, CA, USA), N2 supplement (Gibco Invitrogen, Carlsbad, CA, USA), B27 supplement (Gibco Invitrogen, Carlsbad, CA, USA), 2 mM GlutaMax (Gibco Invitrogen, Carlsbad, CA, USA), and 25 U/ml penicillin/streptomycin (Cambrex, Verviers, Belgium) in the presence of basic fibroblast growth factor (4 ng/ml, FGF, Sigma-Aldrich, St. Louis, MO, USA) in neurosphere culture. Next, 10–15 small aggregates dissected from neurospheres (50,000–150,000 cells in total) and plated to MEA or dissociated into single cell suspension using TrypLe Select (Sigma-Aldrich, St. Louis, MO, USA) and thereafter plated on MEA dishes. The dishes were coated with polyethyleneimine (0.05 % solution, Sigma-Aldrich, St. Louis, MO, USA) and subsequently with mouse laminin (20 μg/ml, Sigma-Aldrich, St. Louis, MO, USA). Differentiation medium supplemented with FGF (4 ng/ml) and brain-derived growth factor (5 ng/ml, BDNF, Gibco Invitrogen, Carlsbad, CA, USA) was replaced three times a week for the MEA cultures. All the MEAs with cells were kept in an incubator (+37 °C, 5 % CO_2_, 95 % air) prior to and between recordings. All recordings were made using MEAs and equipment purchased from Multi Channel Systems (MCS GmbH, Reutlingen, Germany). Figure 1 shows the neuron distribution on 7, 12, and 19 days in vitro (DIV) in MEAs. In addition, cultures grown on the cell culture well plates, were stained with Gamma-aminobutyric acid (GABA) antibody (Rabbit anti-GABA IgG, 1:1000, Sigma Aldrich, St. Louis, MO, USA). Cells were calculated from at least two wells, at least five images and repeated at least twice for each different measurement time point. Additionally, a portion of the cultures were stained either with neuronal marker Mouse anti-$\beta$-tubulin $III$ IgG (1:1200, Sigma Aldrich, St. Louis, MO, USA), with GABA synthesizing enzyme glutamate decarboxylase Mouse anti-GAD67 IgG (1:100, Chemicon International Inc., Temecula, CA, USA) or with calcium-binding protein calretinin Rabbit anti-calretinin IgG (1:800, Swant, Marly, Switzerland). The immunocytochemical protocol has been published previously [9]. hESC experiments were performed at the Institute of Biomedical Technology (University of Tampere, Tampere, Finland). Approval was given to culture the hESC lines (Skottman, R05116) by the Ethics Committee of the Pirkanmaa Hospital District.

### Electrophysiological recordings

Electrical activities were recorded using two 1-well (60MEA200/30 Ti, datasets #8 and #9) and eight 6-well MEAs (60-6wellMEA200/30iR-Ti-w/o; all from MCS GmbH, Reutlingen, Germany). All MEAs had internal reference electrodes. Signals were sampled at 20 or 50 kHz, and stored on a standard PC using MC Rack software (MCS GmbH, Reutlingen, Germany). During the measurements, the culture temperature was maintained at +37 °C using a TC02 temperature controller (MCS GmbH, Reutlingen, Germany). Recordings were visually inspected for artifacts and the measurements or channels likely to contain artifacts were excluded from further analysis.

MEA recordings from ten hESC-NNs were used with an approximated spike train (sequence of spikes) length of 300 s. The hESC-NNs were measured as follows: the first measurement time point was at 7 DIV when the neurons in at least 10 % of the channels of the MEA were active, and when at least 100 spikes within the 300 s were found in the active channels during the recording period. To make the hESC-NN datasets #1–#10 comparable, they were grouped according to the measurement time points (MTP) 1–6, which correspond to 7–26 DIV (see Table 1). The spontaneous activity developed by the hESC-NNs is important in neural development and includes differentiation, maturation, and generation of neuronal processes and connections [6, 9]. Channels were considered as inactive when less than 20 spikes/min [10] were recorded at the last measurement time point (measurement time point 5 or 6). Additionally, if less than two channels per well were active, the well data were excluded from further analysis.

To get a reference for the simulation, we calculated the medians and lower and upper quartiles of spike rate, burst rate, burst duration, and average number of spikes per burst separately for all electrodes and all measurement time points, as shown in Fig. 3. Briefly, the burst analysis algorithm, which was used to examine the intrinsic bursting, relies on the cumulative moving average (CMA) and the skewness ($\alpha$) of the interspike interval (ISI) histogram. For bursting, the ISI threshold was found at the ISI closest to the value of $\alpha \cdot CMA_m$, where $CMA_m$ is the average of *CMA*. Additionally, three or more spikes had to be in a row. The CMA algorithm does not use a fixed ISI but adapts to the dynamics of the studied spike trains. Burst duration means the time between the first spike’s peak and the last spike’s peak. Kapucu et al. [10] have demonstrated the functionality of the tool for highly variable network structures and time-varying dynamics such as in hESC-NNs. In 78 % of all electrodes, the spike rate increased from measurement time point 1 to measurement time point 5. In 16 % of electrodes it decreased, and in 6 % it remained stable or zero. In 70 % of all electrodes, the burst rate increased from measurement time point 1–6. In 20 % of electrodes it decreased, and in 10 % it remained stable or zero. The datasets showed a large variability. For model validation, the means of spike rate and burst rate per well were calculated. The wells were grouped according to the spike rate at measurement time point 5 in low (<50 spikes/min), medium (between 50 and 250 spikes/min), and high (>250 spikes/min) activity (Table 2). This is a kind of normalization to be able to compare the measurements. To get some similarity of the varying cultures, we used only the medium activity datasets for the analysis and simulations. Figure 3 displays the development of neuronal network activity in the medium range. Even if the spike rate and the burst rate showed high variability, the general tendency in both features is an increase.

**Table 1:** Sorted measurement time points (MTP) of the cultured hESC-NNs

Dataset number	MTP 1	MTP 2	MTP 3	MTP 4	MTP 5	MTP 6
#1	7	10	14	17	23	×
#2	7	10	14	17	23	×
#3	7	12	×	19	21	24
#4	7	12	×	19	21	24
#5	7	12	14	×	22	26
#6	7	11	14	18	22	×
#7	7	11	14	18	22	×
#8	7	9	16	×	22	25
#9	7	9	16	×	22	25
#10	7	12	×	×	21	25

The sign × means no measurement was done on this measurement time point. The first MTP was on the 7th day in vitro (DIV). MTP 2 was between 9 and 12 DIV, MTP 3 was between 14 and 16 DIV, MTP 4 between 17 and 19 DIV, MTP 5 between 21 and 23 DIV, and MTP 6 between 24 and 26 DIV

**Table 2:** The table below indicates the number of wells with corresponding activity

Dataset number	Low	Medium	High
#1	0	3	3
#2	1	3	0
#3	0	1	0
#4	1	2	0
#5	1	0	0
#6	1	2	0
#7	1	2	0
#8	1	0	0
#9	0	1	0
#10	2	3	0
Sum	8	17	3

Measured data on each well were grouped for all ten datasets according to the spike rate on MTP 5 in low (<50 spikes/ min), medium (between 50 and 250 spikes/ min), and high (>250 spikes/ min) activity. Datasets #8 and #9 are recorded with 1-well MEAs; all others with 6-well MEAs

### INEX model

To simulate the maturing hESC-NN, we used our spiking neuronal model called INEX [24]. Briefly, the phenomenological model is a cellular automaton whose cells are neurons with two possible states: ON or OFF. Each neuron obtains several inputs and produces exactly one output (spike or no spike). In order to simulate spontaneous activity, we assume that the spikes obey an inhomogeneous Poisson distribution [25]. The momentary firing rate $\lambda _i$ of neuron *i* in time slice $t_k$ is calculated as follows:

1 $$\begin{aligned} \lambda _i(t_k) ={\left\{ \begin{array}{ll} c_i + \sum \limits _{j} y_{ji}s_j(t_{k-1}), &{}\quad \text {if } c_i + \sum \limits _j y_{ji}s_j(t_{k-1})>0\\ 0, &{} \quad\text {otherwise} \end{array}\right. }, \end{aligned}$$

where $c_i$ denotes the basic activity (which include all kind of noise sources such as thermal noise), $y_{ji}$ the synaptic strength of all neurons *j* connected to neuron *i* and $s_j$ the particular spike of the previous time slice of neuron *j* (1 for a spike and 0 for no spike). To find appropriated values for the parameters types $c_i$, $y_{ji}^+$ and $y_{ji}^-$, a brute force approach was used. The parameter values were randomly chosen from a triangular distribution. The values lie between zero and an upper boundary that is at most 1. For $c_i$, the upper boundary varies from 0.01, 0.02, …, 0.09, for the excitatory synaptic strength $y_{ji}^+$ from 0.1, 0.2, …, 0.9 and for the inhibitory synaptic strength $y_{ji}^-$ from −0.1, −0.2, …, −0.9. For the evaluation of the parameter space search, the mean values of the basic activities and synapses strengths of all neurons were calculated. The objective functions of the parameter space search are the spike and burst rate obtained from the experimental data. This means that they are approximately in the range of the MEA data (see Table 3). The brute force method was applied to the simulated data of each virtual measurement time points (vMTP). The vMTPs are considered to resemble the actual measurement time points.

The probability $P_i$ for the occurrence of a spike in time slice $\Delta t$ is defined as follows:

2 $$\begin{aligned} P_i(1 ~spike ~in ~\Delta t) = \exp (-\lambda _i\Delta t)\cdot (\lambda _i \Delta t). \end{aligned}$$

The time slice $\Delta t$ is chosen with a length of 5 ms to cover the temporal length of the action potential and the subsequent refractory period. For each time slice, the algorithm tests if $x_i<P_i$, where *x* are uniformly distributed random values. A spike time history was added so that *x* decreases by factor $f = 0.1$ when a spike in the last time slice occurred. This history method corresponds biologically to an enforced state of excitation of a neuron after spiking and ensures synchronous bursting of the neurons. To summarize, our model has four parameters: $c_i$, $y_{ji}^+$, $y_{ji}^-$, and *f*.

### Simulation of maturing neuronal networks

In our in vitro MEA experiments with hESC-NN, circa 50,000 to 150,000 cells were plated on each well. Based on calcium imaging assessment (data not shown) an estimated 1000–4000 neurons were active and could be recorded. Based on these findings, we chose to simulate 1000 neurons. In the MEA data, one electrode signal is the sum of activity of possible one or several neurons detected by the electrode. In the INEX model, we can consider that one computational neuron corresponds to the activity shown by one electrode. Thus, the model depicts the activity seen by the measurement system as in many other neuronal network models [22, 23]. In the brain, the common proportion of excitatory pyramidal cells and inhibitory interneurons is considered to be 80 and 20 %, respectively [26]. The inhibitory interneurons are mainly GABAergic neurons (reviewed by Chattopadhyaya et al. [27]). The proportion of GABAergic cells in hPSC-derived neuronal cultures has not been studied to any great extent but, based on the immunocytochemical analysis, the portion of GABA positive cells varies between 35 and 90 %, depending on the differentiation method used [28–30]. Here, we performed GABA analysis of cultures paralleling the measurement time points. The portion of GABA positive cells varied between 13 and 19 % of the total neuronal cells (Fig. 4). Thus, for the simulation model, we used the common proportion of 80 % of excitatory neurons and 20 % of inhibitory neurons.

We assumed that there are no connections between neurons on the day of plating and no autapses [31, 32], which are self-connections of a neuron. The INEX model only allowed the addition of connections. Therefore, no reduction of connections [11] was simulated. Connections appeared simultaneously between two sequential vMTPs. The model did not take into account apoptosis or proliferation, and we did not include transmission delays or cell movement in the model.

In order to model the maturing process and the developing connectivity of the neural network, we started with a few randomly chosen connections with a probability of 1 % of all possible connections and weak synaptic strength for vMTP 1, respectively. Thus, the neuronal network was not inactive at the first simulation step (vMTP 1). Then, the connection probability was increased to 2, 4, 6, 8 %, and up to 10 % of all possible synaptic connections (corresponding to vMTP 2 to vMTP 6) [22]. The 10 % connection probability corresponded to the connection probability in matured neuronal networks. The arrangement of connections between the neurons was selected randomly. For every vMTP, the connections in the simulated neuronal network were redefined. The values of the synaptic strengths were automatically varied with a brute force approach, as presented above. Additionally, we simulated according to the following scenario: (1) an increase of the activity between vMTP 1 and vMTP 6; (2) an increase of the activity between vMTP 1 and vMTP 5, and a decrease at vMTP 6, as seen in Fig. 3. All resulting spike trains had a length of 300 s. The simulation tool was then run ten times with these constraints to get statistically significant data.

### Validation of the simulated spike trains

For validation, we calculated four features [spike rate (spikes/minute), burst rate (bursts/minute), burst duration (in seconds), and the average number of spikes per burst] for each of the simulated spike trains using the burst analysis tool described by Kapucu et al. [10]. The results were then compared with the same features obtained from the ten previously mentioned MEA experiments with hESC-NNs. The spike rate and burst rate were selected as goal functions for the parameter search. Too many features would lead to an over-fitting, and thus produce unstable points. The other two parameters, burst duration and average number of spikes per burst, described the burst structure and seemed to undergo typical changes during the maturation of the network.

## Results

As a basis for our simulations, we conducted 10 MEA experiments (two 1-well MEAs each with 60 electrodes and eight 6-well MEAs each with nine electrodes) with hESC-NNs. The datasets were grouped according to six measurement time points that correspond to 7–26 days in vitro in MEAs (Table 1). The INEX model generated a large-scale network of 1000 neurons that corresponds to the number of active cells in the experiments with hESC-NNs. For the vMTP 1–6 used in the simulations, we created a neuronal network with increasing connection probability over time. We applied a brute force method to each obtained dataset to find one parameter set (comprising the basic activity, the excitatory and inhibitory synaptic strengths, and a factor for the spike time history) that produced neuronal activity that best resembled the experimental data.

We kept the basic activity, which was modeled as the random noise of each neuron in the system, as constant as possible for vMTP 1–6 with the hypothesis that during maturation only the network properties will change. Thus, only the inhibitory and excitatory synaptic strengths were more variable (in comparison to the basic activity which remains stable over the measurement time points). The simulated network showed an increase in the excitatory synaptic strengths over time (Table 3). This increase continued until the final vMTP where a decrease in excitatory synaptic strengths was observed. The inhibitory strengths remained stable over the simulated time duration. For each vMTP, we simulated ten datasets, each with 1000 neurons. For the first nine neurons (corresponds to the number of electrodes on a 6-well MEA), we calculated the lower and upper quartile as well as the median of four features, in particular spike rate, burst rate, average number of spikes per burst, and burst duration. Table 3 and Fig. 3 show both the development of the four features from measurement time point 1–6 for both experimental and simulated data. The validation showed that all calculated median values of the spike rate in the INEX data are within the lower and upper quartile of the MEA data. This was also the case for the burst rate with the exception of vMTP 6. Nevertheless, the upper quartile of the simulated data was within the quartile range of the experimental data. In three out of six measurement time points, the median and quartiles of the burst duration in the simulated data were higher than in the MEA data. The median of the average number of spikes per burst was mostly within the quartile range of the experimental data. For the spike and burst rate as well as for the average number of spikes per burst, we saw an increase in the features over time in the experimental data and correspondingly in the simulated data. The spike rate and burst rate dropped at the last measurement time point in the experimental, and thus also in the simulated data. The alternating burst duration over maturation can be seen in both the experimental and simulated data.

**Table 3:** Lower quartile (Q1), median (M) and upper quartile (Q3) of the calculated features for simulated (INEX) and experimental (MEA) data on measurement time point (MTP) 1–6

Data	Feature	MTP 1			MTP 2			MTP 3		
		Q1	M	Q3	Q1	M	Q3	Q1	M	Q3
MEA	SR	2.71	9.53	36.26	8.11	16.99	49.30	14.92	39.45	144.64
	BR	0.07	0.26	1.08	0.09	0.39	0.99	0.88	1.42	5.22
	BD	0.87	5.25	12.65	1.50	2.50	9.65	2.05	6.98	7.55
	SB	1.57	3.16	4.96	2.00	5.00	11.40	3.08	6.33	7.62
INEX	SR	10.98	11.39	11.98	14.19	15.67	17.74	23.04	24.17	26.15
	BR	0.28	0.40	0.57	0.40	0.47	0.62	0.42	0.60	0.84
	BD	13.99	18.09	21.43	6.85	10.17	27.46	11.87	16.56	18.36
	SB	4.15	5.61	6.38	4.02	4.56	10.95	7.00	9.01	10.47
Parameter		${c}$	${y_{ji}^+}$	${y_{ji}^-}$	${ c}$	${y_{ji}^+}$	${y_{ji}^-}$	${ c}$	${y_{ji}^+}$	${y_{ji}^-}$
		0.07	0.1	−0.1	0.08	0.1	−0.1	0.08	0.3	−0.1

Data	Feature	MTP 4			MTP 5			MTP 6		
		Q1	M	Q3	Q1	M	Q3	Q1	M	Q3
MEA	SR	57.10	91.21	163.21	109.76	148.48	224.46	53.68	106.90	113.65
	BR	0.89	4.19	7.19	4.29	5.66	10.69	2.24	2.59	4.79
	BD	0.97	2.79	4.45	2.35	3.71	16.23	1.44	12.68	17.97
	SB	4.16	7.00	19.31	12.18	18.67	45.68	7.19	20.00	24.68
INEX	SR	56.02	57.16	60.39	95.83	100.71	104.10	49.64	54.16	56.28
	BR	2.28	2.67	3.15	4.60	5.34	6.26	1.63	2.06	2.46
	BD	10.05	11.14	12.65	4.18	5.13	5.93	9.67	12.96	15.75
	SB	13.07	14.94	16.46	11.43	13.37	14.15	12.57	15.14	17.97
Parameter		${ c}$	${y_{ji}^+}$	${y_{ji}^-}$	${ c}$	${y_{ji}^+}$	${y_{ji}^-}$	${ c}$	${y_{ji}^+}$	${y_{ji}^-}$
		0.09	0.5	−0.1	0.09	0.5	−0.1	0.09	0.3	−0.1

The table shows as well the selected upper boundary as result of the parameter space search which resembled best the spike and burst rate of the experimental data (see "Methods" section/ INEX model). The table is visualized in Fig. 3

The spike rate *SR* is given in spikes per minute, the burst rate *BR* in bursts per minute, the burst duration *BD* in seconds and the average number of spikes per burst *SB* as count.

**Fig. 2:**
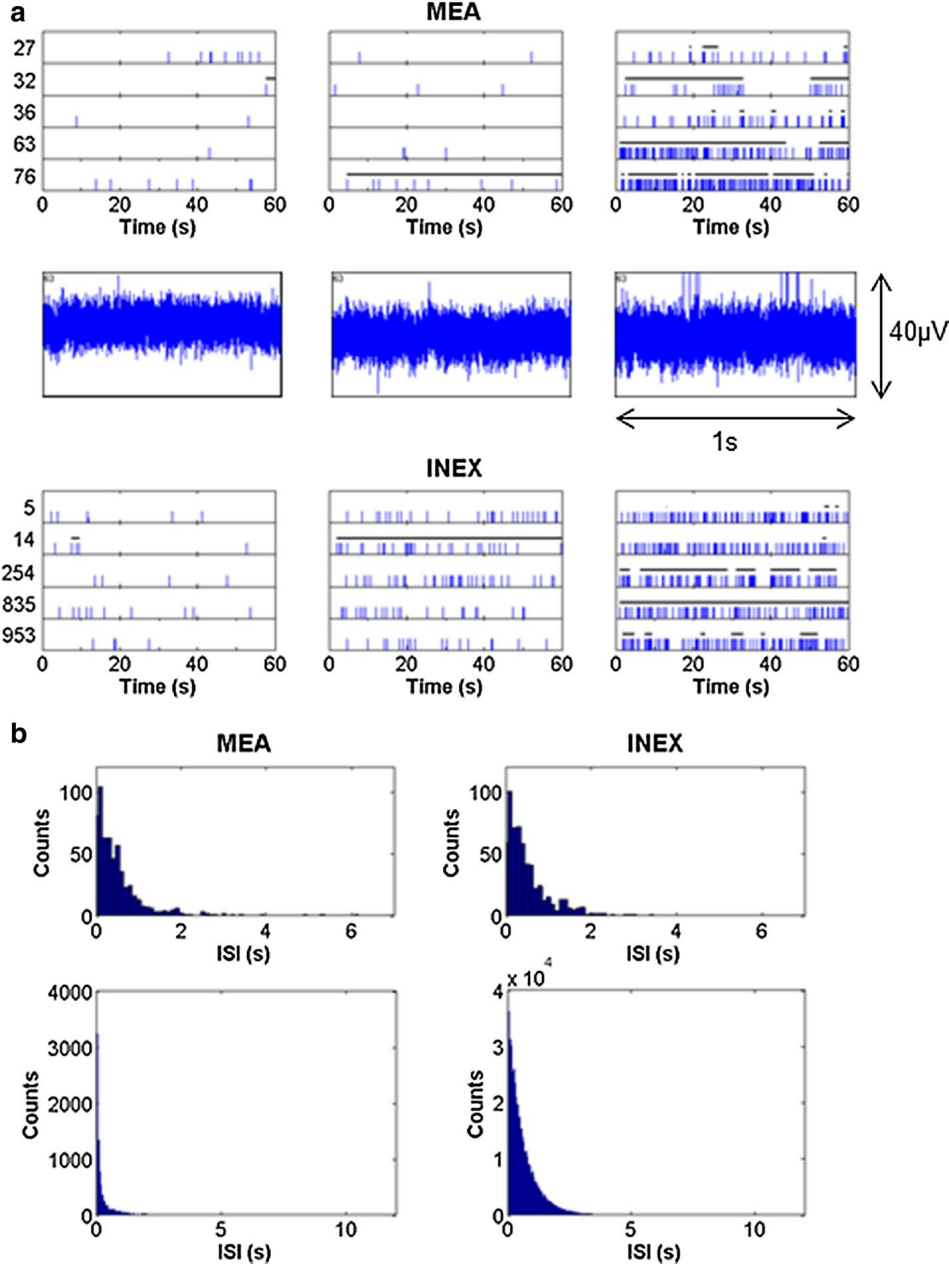
Comparison of spike trains and ISI histogram of both the experimental and simulated data. **a** The *upper row* shows snippets of example spike trains of the measured hESC-NNs at five electrodes of dataset #9 (electrode number on the *y* axis). The *middle row* shows the raw voltage traces of channel 63. The *lower row* represents the resulting spike trains of five simulated neurons. *Each row* shows measurement time point 1, 3 and 5, respectively. The length of the detected bursts is indicated as* bars* on* top* of the spikes. **b** The *upper row* shows the ISI histogram of one channel/ neuron. On the *left*, an ISI histogram of channel 63 at measurement time point 5 (22 DIV). On the *right*, an ISI histogram of a simulated neuron at vMTP 5. The *lower row* shows the population ISI histogram of dataset #9 at MTP 5 on the *left* and the population ISI histogram of the neuronal network at vMTP 5. Note that we compare the ISIs of 20 active MEA electrodes where the exact number of recorded neurons is unknown with ISIs of 1000 simulated neurons. Thus, the absolute number of spikes cannot be compared and the main information is in the distribution of the histogram

**Fig. 3:**
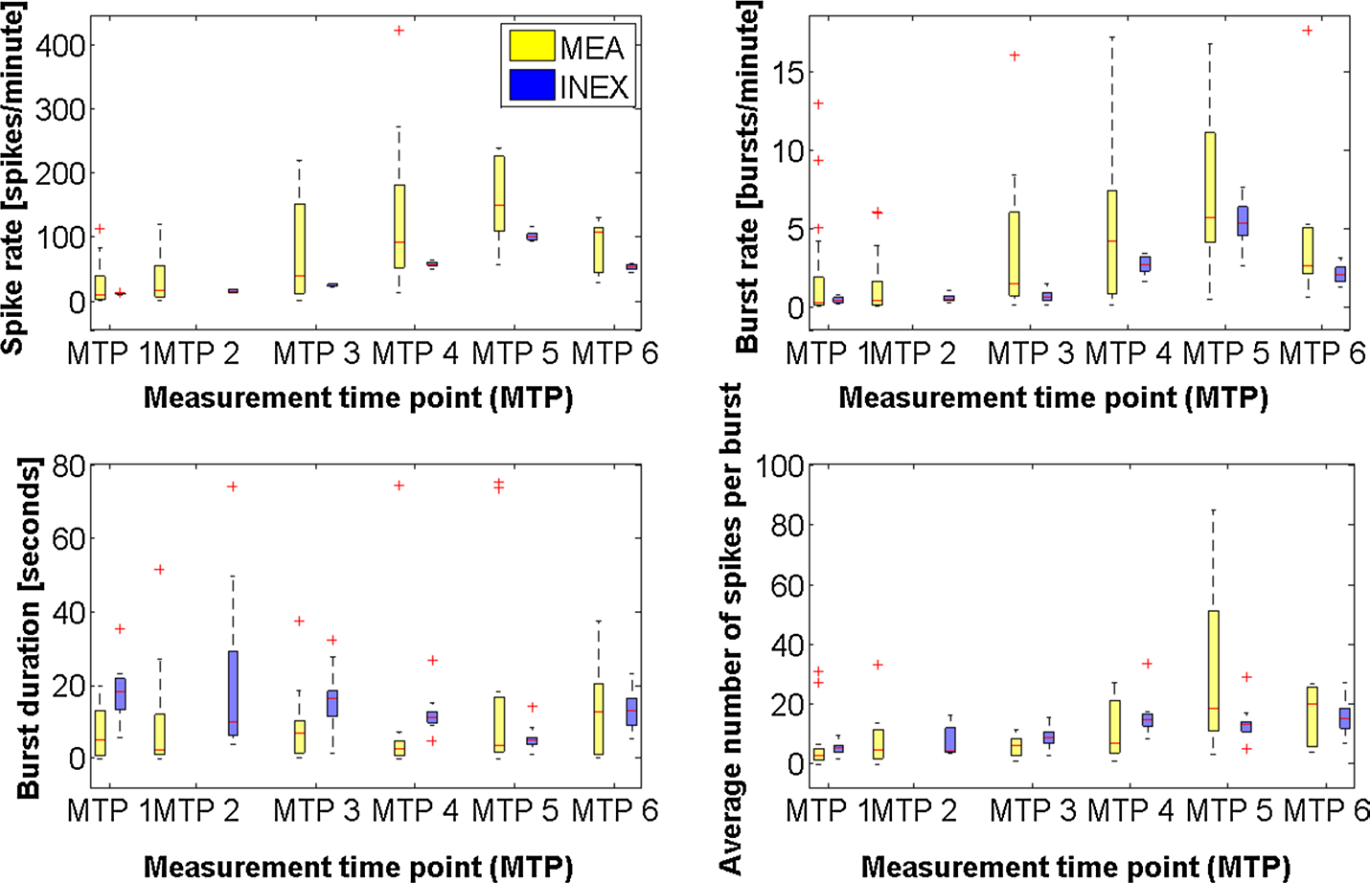
Development of the neuronal activity over time (measurement time point 1–6). Clockwise: medians and quartiles of the spike rate, the burst rate, the average number of spikes per burst and the burst duration of all wells in the medium activity class, respectively. Note that some outliers are not shown in the last two graphs for visibility reasons. The values of each box plot are represented in Table 3

The spike trains of five sample electrodes and five example neurons are displayed in Fig. 2a. The experimental and simulated spike trains of the first measurement time point showed just a few spikes. The overall number of spikes increased with the number of connections and with the number of measurement time points (Figs. 2a,  3). The simulated activity of the last measurement time point exhibited typical spike and burst patterns as recorded from hESC-NNs (see Table 3) [6]. Partly synchronous spiking and intrinsic bursting was recorded for maturated hESC-NNs and could also be seen in the corresponding simulated spike trains. Figure 2a also displays the raw voltage traces of channel 63 of the same hESC-NN. Figure 2b shows the ISI histograms of one experimental and one simulated neuron at measurement time point 5. Both histograms show a similar ISI distribution. By varying the inhibitory and excitatory parameters, the model produced similar spiking characteristics to those measured. Figure 2b displays also the population ISI histograms of dataset #9 and one simulated neuronal network at (v)MTP 5.

## Discussion

### Stem cell data

The potential of human pluripotent stem cells and their neural derivatives in the fields of neurotoxicity, drug screening, developmental biology, and tissue engineering is well known [1, 2, 33]. In these applications, stem cells need to be differentiated into pure neuronal populations and show neuronality in genotype and phenotype as well as at the functional level [33]. Thus, it is also important to study these cells in vitro at the functional level [34]. MEAs are used for the characterization of the network activity of these cells as well as for studying drug and neurotoxic effects on the cells [6, 8]. However, little is known about the development of the network processes that generate the signaling patterns in hESC-NN. Earlier, Heikkilä et al. [6] observed single spike activity in hESC-NN cultured on MEA in the first week followed by the development of spike trains during the next two weeks. From the fourth week onwards, they observed synchronous bursts. Our study had similar results (see spike trains and voltage traces in Fig. 2 and the statistics in Fig. 3) with the exception that the data points used were up to 26 DIV, and thus later points of network maturation were not studied. Here, as a larger data set was analyzed, we found quite high variability in the spike and burst behavior across network maturation. The observed variability can be explained by the different number of cells in the networks and the various fractions of neuronal and glial cells on these spontaneously formed neuronal networks. Furthermore, there is evidence that the neuronal networks are not fully matured even at measurement time point 5 or 6, which corresponds to 21–26 DIV, respectively, and that the networks we used are still in different developmental stages [6, 35], since the signaling of these measurement time points differ from others in terms of both spike and burst behavior.

**Fig. 4:**
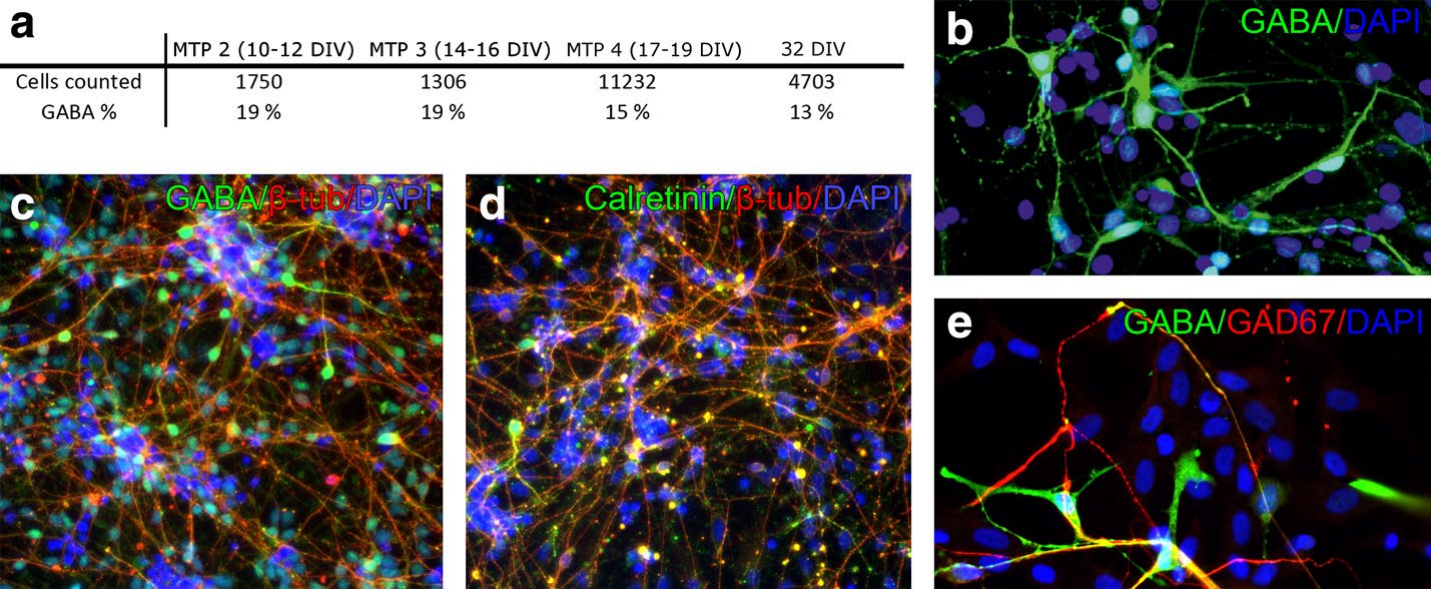
**a** Proportion of GABAergic cells in neuronal population analyzed at different measurement time points (*MTP*). Standard deviations for calculated GABA-positive cell percentages in measurement time points 2, 3, 4, and 32 days in vitro (*DIV*) are 17, 9, 13 and 10 %, respectively. **b** Representative image of GABA-positive cells. **c** Representative image of neuronal network double-labeled with GABA. **d** Cells expressing calcium binding protein Calretinin form a subpopulation of GABAergic cells. **e** Expression of GABA and GABA synthesizing enzyme glutamate decarboxylase labeled with GAD67 define GABAergic neurons. Nuclei (*blue*) are stained with DAPI. The used magnification for **b** and **c** is ×10 and for **d** and **e** ×20

In addition to synaptic activity, several other activity pathways exists especially during development [36]. Especially, gap junction mediated activity is important during development [37] and has been also studied in dissociated neuronal networks cultured on MEAs [20]. In this work, we focus only on synaptic mediated activity, which exists in these human neuronal cultures as proved with pharmacological modification of neurotransmitter receptors [6].

For the burst analysis, we did not use the traditional burst analysis approach with fixed ISI that had been used earlier with similar cultures (e.g., Heikkilä et al. [6]). As Kapucu et al. [10] demonstrated, the traditional approach quite often fails when examining hESCs. Thus, the authors developed the cumulative moving average approach that adapts the ISI threshold for bursts to the network behavior [10]. The method also finds statistically burst-like behavior in spike data from spike trains with quite low firing activity. Here, we use the CMA tool for analysis on both simulated and measured data, resulting in comparable statistical data. The synchronous population bursts behavior described earlier by Heikkilä et al. [6] was not taken into consideration because the used data sets did not cover later time points (1 month onwards).

The field of in vitro experiments with hESC-NNs is quite new and not all of the previously conducted experiments were suitable as a basis for our simulations because we modeled the maturation over a relatively long time period. Even with a limited number of data sets, we can see the tendency of first an increase and later a decrease in neuronal activity, especially in the spike and burst rate (see Fig. 3). Johnson et al. [38] also report that the neuronal activity is reduced during the course of the maturing process.

The in vitro cultures are meant to mimic the neuronal network in vivo. Even the in vitro developed neuronal network might lack certain network structural functions as seen in brain and possible effect, like the electrical field effect, between the neurons might not be observed in the cultured neurons [39]. However, the hESC-NNs provide us a way to model in vitro the human neuronal system that has bot been available earlier.

### Simulation

The INEX model is a very simple, general and flexible model. Despite its primary application for cortical culture modeling [24], it is not bound only to the simulation of cortical networks in vitro. In this study, we use large-scale networks with 1000 neurons to study spike and burst behavior in hESC-NNs. Here, the neurons are considered as points with neither spatial extension nor bio-physical structure (no axons, soma, or dendrites) and the connections representing synapses are formed randomly between these virtual neurons. We made a number of simplified assumptions which are described in the  "Methods" section. There is evidence that neurons are interacting with synapse communication, with gap junction mediated exchange of ions and small molecules, like ATP (adenosine triphosphate), and with electric field effects [40]. Computational models of neuronal networks simulate the synaptic transmission per se; however, we can consider that the model of interaction includes all communication as the parameters of the synaptic interaction models are tuned to provide similar responses as in the actual biological networks. Further, as the communication through the other pathways is not directly mediated by spiking activity modeling such weak and less known pathways, it is not considered the core of the present study. It has been previously shown that these networks and cultures have a minority of astrocytes [9]. The INEX model does not take glial cell effect directly into account. However, the effect is inbuilt with the effect of the spike history.

It is assumed that synapses develop during the maturation process, and that mature in vitro networks have a connectivity of about 10 %. This means that each neuron is connected to 10 % of the other neurons. For the simulation, the starting point is almost no connectivity (1 %), and the end point has 10 % connectivity [22]. The steps in between correspond to virtual measuring points and are determined linearly (1, 2, 4, 6, 8 and 10 %). In line with this, the experimental measuring points are also almost linear. Another approach would be to increase the connectivity exponentially with restricted resources as described by Lai et al. [41]. However, a detailed connectivity analysis of hESC-NN has not yet been carried out. Therefore, we did not follow this approach in this paper.

Present technology such as MEA or patch clamp cannot provide the connectivity analysis reliably, the results from the INEX model strengthen the concept that the maturing hESC-NN and its spiking activity can indeed be explained by the development of connectivity between the neuronal cells. In biological networks, the development of connectivity can be generally explained either as increased synaptic strengths, increased number of synapses between the processes, or an increased number of processes between cells [6, 14]. Nevertheless, an overproduction of synaptic connections is followed by the elimination of some synapses and the stabilization of activity [14]. The results indicate that the model can simulate the reduction of synapses [42, 43], which is an important feature of the maturating process, by changing the synaptic strengths. Thus, the number of neurons remains the same over all virtual measurement time points. Without thorough biological characterization of the time course of this development in vitro, the separation of these processes using model concepts is in practice very difficult or even impossible. Therefore, these difficulties must be taken into account when evaluating the results presented in this paper.

A steadiness or increase in the excitatory synaptic strengths is seen in the simulations from vMTP 1 to vMTP 5. At vMTP 6, the excitatory strengths are slightly reduced and result in reduced spike and burst activity, as seen in the experimental data. The inhibitory synaptic strengths, however, remain stable over time. From the simulations, we can draw the conclusion that the share of inhibitory neurons is relatively low, as the inhibitory strengths remain low. This can also be observed in the experimental data (see Fig. 4). Moreover, if the proportion of the inhibitory and excitatory neurons is incorrect, the strengths and ratio of excitatory and inhibitory neurons in the simulation can compensate for this situation. As both strengths and the number of inhibitory neurons remain low, we consider the conclusions to be correct. The calculated features adapted from spikes and bursts show that the maturing process of hESC-NNs can be modeled by growing connectivity in the simulated network (Table 3). The ISI histograms of one neuron in the experiments and in the simulation match very well. In the population ISI histograms, we also see an exponential distribution but more bins with small ISIs in the simulated data (Fig. 2b). This can be explained by the fact that the INEX model produces more regular bursts with short ISIs.

Burst duration for simulated and experimental data differ slightly (Table 3; Fig. 3). From measurement time point 4 to measurement time point 5, the burst duration in the experimental data increases, whereas it decreases in the simulated data. We assume that the burst duration in the INEX model is mainly determined by the inhibitory strengths. We would like to stress that choosing the spike rate alone as objective function leads to non-unique parameters sets in the fitting process. Therefore, additional objective functions like burst rate have to be chosen.

Our model uses a spike time history that provides some adaptation and control based on previous time instances. However, the simplicity of the spike time history implementation is not suitable for modeling short-term plasticity. Moreover, we did not model the long-term plasticity over measurement time points because each measurement time point was modeled by an explicitly tuned network. Gritsun et al. [15] showed that long-term plasticity does not play a role when explaining burst properties in the first three weeks of development. Making a plasticity-based neuronal network development model would require a large number of data points in the experimental data as well, which is not the case in this work.

## Conclusions

To summarize, we present a computational model of hESC-NNs and their maturation for the first time. The simulations show that the maturing process of the network, which is modeled by the increased connectivity reflecting the formation of new synapses and connections to other neurons, can explain the spike characteristics and appearance of bursts during maturation. In other words, our model, based on the assumption that there is an interaction between excitatory and inhibitory neurons, explains that the maturation of a neuronal network and the spontaneous emergence of bursts are due to the formation of synapses. Our model and its future development, which includes topology of the developing connectivity, has the potential to improve our understanding of the maturing process of hESC-NNs.

## Abbreviations

ATP: adenosine triphosphate
BDNF: brain-derived growth factor
CMA: cumulative moving average
GABA: gamma-aminobutyric acid
hESC: human embryonic stem cells
hESC-NN: human embryonic pluripotent stem cells-derived neuronal networks
hPSC: human pluripotent stem cells
INEX: model called ”INhibitory-EXcitatory”
ISI: interspike interval
MEA: microelectrode array
MTP: measurement time points
vMTP: virtual measurement time points
